# Relationship between fluorescence yield and photochemical yield under water stress and intermediate light conditions

**DOI:** 10.1093/jxb/ery341

**Published:** 2018-10-08

**Authors:** Xuejuan Chen, Xingguo Mo, Shi Hu, Suxia Liu

**Affiliations:** 1 Key Laboratory of Water Cycle and Related Land Surface Processes, Institute of Geographic Sciences and Natural Resources Research, Chinese Academy of Sciences, Chaoyang District, Beijing, China; 2 University of Chinese Academy of Sciences, Shijingshan District, Beijing, China; 3 College of Resources and Environment/Sino-Danish Center, University of Chinese Academy of Sciences, Shijingshan District, Beijing, China

**Keywords:** Chlorophyll fluorescence, intermediate light, leaf scale, pulse-amplitude modulation, photochemistry, summer maize, water stress

## Abstract

The dynamics between fluorescence (F_s_) yield and photochemical (P) yield in a changing environment are essential for understanding the relationship between photosynthesis and fluorescence. The ratio of F_s_ yield and P yield tends to be constant under high light intensity, but the relationship between these two yields, and its response to environmental conditions, need to be explored further under intermediate and low light. In this study, we performed leaf-scale measurements of fluorescence parameters by pulse-amplitude modulation (PAM) technology in summer maize (*Zea mays* L.) plants grown under intermediate light conditions in a climate chamber. Plants were treated as moderately water stressed and non-water stressed. Results showed that a decrease in P yield was accompanied by increases in F_s_ yield and non-photochemical quenching (NPQ) yield in response to moderate water stress under intermediate and low light conditions. F_s_ yield was negatively correlated with P yield under intermediate and low light conditions when there was sufficient soil water in the root zone. Under water stress, the correlation between F_s_ yield and P yield was negative in low light, but became positive under higher light levels. F_s_ yield was negatively related to P yield when NPQ yield was low; however, they were synergistically and positively associated when excessive light dissipation was dominated by NPQ.

## Teaser

Under intermediate and low light conditions, the relationship between fluorescence yield and photochemical yield in water-stressed C_4_ leaves is non-linear. Non-photochemical quenching yield regulates this relationship.

## Introduction

Chlorophyll fluorescence is red and far-red light (650–800 nm) emitted by chlorophyll *a* pigments a few nanoseconds after light absorption (Porcar-Castell *et al.*, 2014; Damm *et al.*, 2015; Verrelst *et al.*, 2016). It is sensitive to short-term environmental stresses such as drought or nutrient deficiency, among others. (Flexas *et al.*, 2002; Souza *et al.*, 2004; Dobrowski *et al.*, 2005; Zarco-Tejada *et al.*, 2013; Cendrero-Mateo *et al.*, 2015; Tubuxin *et al.*, 2015; Chen *et al.*, 2016). Chlorophyll fluorescence has been used as a non-destructive and non-intrusive probe in studies of plant photochemistry, physiology, and ecology (Flexas *et al.*, 1999; Lee *et al.*, 2013; Guanter *et al.*, 2014; Cendrero-Mateo *et al.*, 2015). Remotely sensed chlorophyll fluorescence from satellite imaging is providing new data sources to characterize the dynamics of gross photosynthesis at regional scale, increasing our ability to monitor and assess the effects of environmental stresses on the productivity of vegetation (Lee *et al.*, 2013; Guanter *et al.*, 2014; Wang *et al.*, 2016; Zhang *et al.*, 2016*a*; Yang *et al.*, 2017).

Studies have shown that solar-induced fluorescence (SIF) is closely related to gross primary production (GPP) at regional or ecosystem scale (Guanter *et al.*, 2014; Zhang *et al.*, 2016*a*), especially when there is water stress (Lee *et al.*, 2013; Wang *et al.*, 2016). It has been reported that SIF and GPP are consistent in terms of spatial patterns and seasonal dynamics (Guanter *et al.*, 2014; Yang *et al.*, 2015; Zhang *et al.*, 2016*a*; Yang *et al.*, 2017). SIF traces the variations of GPP much better than the enhanced vegetation index, normalized difference vegetation index, and land surface water index do (Lee *et al.*, 2013; Wagle *et al.*, 2016; Wang *et al.*, 2016). For example, during the 2012 drought event in the Great Plains of the USA, it was found that variations of SIF and GPP were consistent, and SIF declined more significantly than the normalized difference vegetation index (Sun *et al.*, 2015; Wang *et al.*, 2016). As SIF is more sensitive to changes in vegetation photosynthesis and water status than greenness indices, it is a better indicator of photosynthesis (Guanter *et al.*, 2014; Yang *et al.*, 2015; Zhang *et al.*, 2016*a*; Yang *et al.*, 2017). So far, some empirical linear relationships between SIF and GPP have been developed to directly estimate the productivity of vegetation using remote sensing of SIF (Guanter *et al.*, 2014; Wagle *et al.*, 2016).

Although some studies have demonstrated close associations between chlorophyll fluorescence and GPP (Guanter *et al.*, 2014; Zhang *et al.*, 2016*a*), the quantitative relationship and inherent mechanisms of the links between them are still not clear. In photosystem II (PSII), chlorophyll fluorescence, photochemical reactions, and non-photochemical quenching (NPQ) are three pathways that consume all of the light energy absorbed by the leaf (van der Tol *et al*., 2014). These three pathways are closely associated, and the sum of fluorescence (F_s_) yield, photochemical (P) yield, and NPQ yield is considered to equal 1 (Hmimina *et al.*, 2014; van der Tol *et al*., 2014; Lee *et al.*, 2015; Cendrero-Mateo *et al.*, 2016; Zhang *et al.*, 2016*b*). F_s_ yield is the fraction of absorbed light energy re-emitted as fluorescence, and represents the light use efficiency of fluorescence (Damm *et al.*, 2010; Lee *et al.*, 2015; Yang *et al.*, 2017). The P yield of PSII, which is the fraction of absorbed photons used for photochemical reactions, provides an estimation of photochemical light use efficiency (Baker, 2008; Lee *et al.*, 2015; Yang *et al.*, 2017). The surplus part of the absorbed light energy is dissipated as heat (i.e. NPQ), including the constitutive thermal dissipation and variable energy-dependent heat dissipation. A simple competition model has been proposed for these three processes (Butler, 1978; Baker, 2008). *In situ* measurements or mechanistic model simulations have revealed that the relationship between fluorescence yield and photochemical yield is not perfectly linear (van der Tol *et al.*, 2009, 2014; Lee *et al.*, 2015; Zhang *et al.*, 2016*b*; Cui *et al.*, 2017) and is influenced by the absorbed photosynthetically active radiation (van der Tol *et al.*, 2009, 2014; Cui *et al.*, 2017). At present, our understanding of the non-linear relationship between the fluorescence and photochemical yields is still insufficient. It has been observed that the ratio of the F_s_ yield to the P yield tends to be constant under high light condition, that is, in the imaging time of satellite SIF (Damm *et al.*, 2010; Guanter *et al.*, 2014; Lee *et al.*, 2015; Liu *et al.*, 2017). However, under intermediate and low light conditions (i.e. the early morning and late afternoon), the relationship between these two yields is not clear, and needs to be further investigated.

Fluorescence and photochemical processes are sensitive to environmental stresses (Flexas *et al.*, 2002; Cendrero-Mateo *et al.*, 2015; Chen *et al.*, 2016). In the context of climate change, droughts have become more frequent and severe around the world in recent decades (Spinoni *et al.*, 2014). Drought has been reported to induce significant decreases of P yield and F_s_, and increases of NPQ (Flexas *et al.*, 2002; Souza *et al.*, 2004; Baker, 2008; Faraloni *et al.*, 2011; Liu *et al.*, 2012; Cendrero-Mateo *et al.*, 2015). However, few studies have explored the effects of water stress on the relationships between these parameters (Flexas *et al.*, 2002; Cendrero-Mateo *et al.*, 2015).

This study explored the fluorescence and photochemical physiology in response to water stress under intermediate and low light conditions. It is hypothesized that the relationship between F_s_ yield and P yield is not perfectly linear, and depends on water status and light intensity. As summer maize (*Zea mays* L.) is one of the most important and widely planted C_4_ crops (in which photorespiration is negligible) in the world, we conducted an experiment with summer maize in a climate chamber with two soil moisture treatments (moderate water stress and no water stress). The fluorescence parameters were measured in leaves by using a pulse-amplitude modulation (PAM) fluorometer. This study aimed to explore (i) the relationship between F_s_ yield and P yield under intermediate and low light conditions, and (ii) the effects of water stress on F_s_ yield, P yield, and the relationship between them. The results provide new knowledge about the interaction between leaf photochemistry and fluorescence, which is crucial for improving the estimation of vegetation productivity.

## Materials and methods

### Description of the experiment

**Fig. 1.**
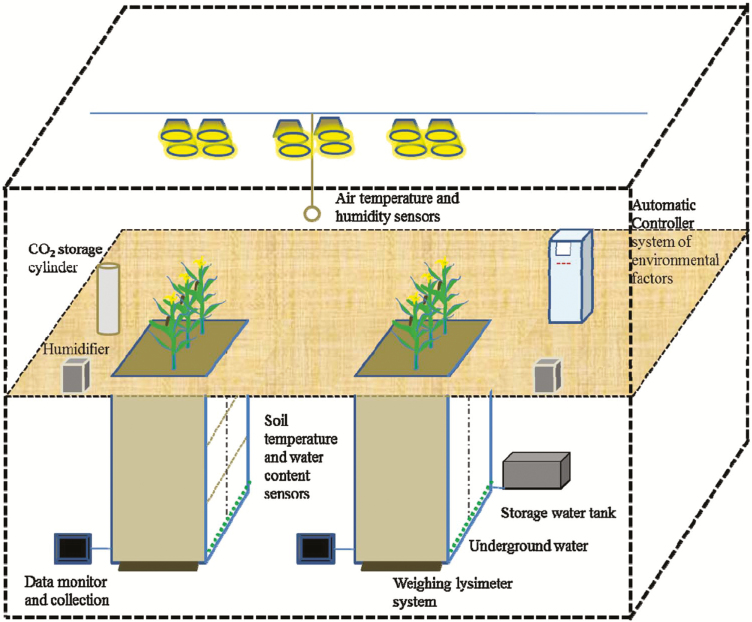
Schematic diagram of the WATDPED (Tan *et al.*, 2017).

This experiment was conducted between 12 October 2016 and 24 February 2017 using the Water Transformation Dynamical Processes Experimental Device (WATDPED; Fig. 1) established in the Institute of Geographic Sciences and Natural Resources Research, Chinese Academy of Sciences, Beijing, China. The WATDPED consists of a climate chamber with two 3 × 2 × 3 m (length × width × height) lysimeters. The lysimeters contain alluvial soil taken from farmlands in the North China Plain, which is composed of 60.22% sand, 39.09% silt, and 0.70% clay. The field capacity is 25.12% and residual moisture is 2.66%. The climate chamber has an artificial light source composed of metal halide lamps and sodium lamps with a maximum illumination of 30000 lx. There are two centrifugal humidifiers to control the humidity and an air conditioning system to control the air temperature.

**Fig. 2.**
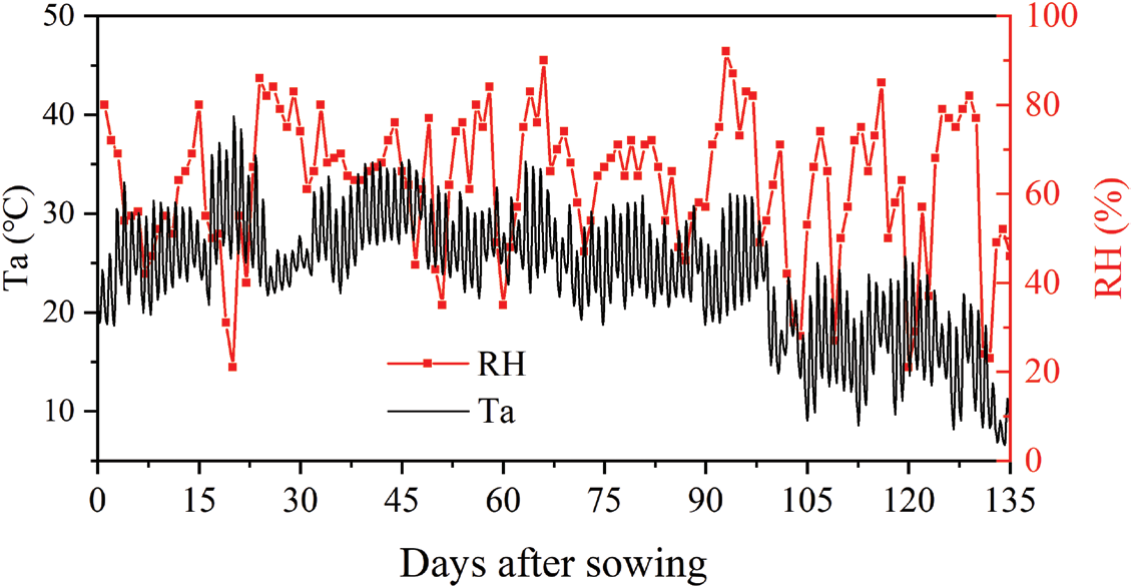
Variations in 3 h averaged air temperature (Ta) and daily relative humidity (RH) during the summer maize growth period. These values were used as the inputs for the climate conditions, controlled by an air conditioner and centrifugal humidifiers, in the climate chamber.

Seeds of summer maize (*Zea mays* L., cultivar JD68) were sown in soil with an intra-row space of 40 cm and inter-row space of 30 cm; there were 36 plants in each lysimeter. According to the photosynthetic light response curve of summer maize (Stirling *et al.*, 1994; Liu *et al.*, 2012), when photosynthetically active radiation (PAR) is greater than ~700 μmol m^–2^ s^–1^, the photosynthetic rate increases slowly with increasing PAR, while when PAR is lower than ~450 μmol m^–2^ s^–1^, the photosynthetic rate increases rapidly with PAR. Thus, PAR in the range of 450–700 μmol m^–2^ s^–1^ was taken as the intermediate light condition and PAR lower than 450 μmol m^–2^ s^–1^ was taken as the low light condition for the summer maize in this study. In order to explore the relationship between P yield and F_s_ yield under intermediate and low light conditions, the light intensity above the canopy was kept at 700 µmol m^–2^ s^–1^ during the whole growth period by adjusting the distance between the canopy top and the lamps. The light source was turned on at 06.00 h and off at 18.00 h automatically, so that the plants were exposed to a 12 h photoperiod. In the climate chamber, variations of 3 h averaged air temperature and daily humidity were set according to records made at the Beijing climatological station (116°28′E, 39°48′N) during the period 16 June to 29 October 2010, to represent normal climate conditions (Fig. 2). Chemical fertilizers were applied at the seedling, early jointing, and tasseling stages (Table 1).

**Table 1.** Records of the growing stages of summer maize and the use of fertilizer during the experiment

Growing stage	DAS (NS)	DAS (WS)	Fertilizer	Amount of fertilizer (g m^–2^)
Seedling	14–29	14–34	Compound fertilizer	40
Jointing	30–59	35–64	Urea	30
Tasseling	60–74	65–82	Urea	40
Filling	75–105	83–115	None	–
Maturity	106–130	116–135	None	–

DAS, days after sowing; NS, non-water stress; WS, water stress.

Plants in the two lysimeters were subjected to two treatments, moderate water stress (WS) and non-water stress (NS). The soil water contents (% by volume) of the root layer (25 cm depth) were monitored automatically by 5TE soil water sensors (Decagon, Pullman, WA, USA) and were maintained at 45–50% of field capacity for the WS treatment and no less than 80% of field capacity for the NS treatment by irrigation. The weight of each lysimeter was recorded automatically at 30-min intervals with a precision of 180 g, and the weights were used to estimate the changes in evapotranspiration (ET). A water stress index (WSI) was calculated as

$$\text{WSI}=\frac{\theta -\theta_{r}}{\theta_{\text{c}}-\theta_{r}}$$ (1)

where *θ* is the measured soil water content of the root layer, $\theta_{r}$ is the residual moisture content, and $\theta_{c}$ is the field capacity. The range of values of WSI is 0–1. When *θ* > $\theta_{c}$, WSI is taken as 1.

For the measurements of growth indicators and fluorescence parameters, five plants in each treatment were randomly selected as biological replicates. Three fully expanded top leaves from each plant were sampled to measure chlorophyll content, and the chlorophyll content was calculated as the mean value of the three measurements. Fluorescence parameters were measured using the third fully expanded top leaf of each selected plant. During the growth period, measurements were made on the same plants, but the top expanded leaves may be updated in different growing stages. The chlorophyll content and fluorescence parameters of summer maize plants were measured in the middle of the leaf at 5-day intervals during the growth period. As we focused on photochemical yield in PSII rather than CO_2_ assimilation, photosynthesis in terms of gas exchange was not measured in this experiment.

### Measurement of growth indicators

Basic growth indicators including plant height, leaf area index (LAI), and relative leaf chlorophyll content were measured. TheSPAD-502 Plus chlorophyll meter (Konica Minolta Sensing Inc., Japan) was used to measure the relative chlorophyll content. The relative amount is an indexed estimation of chlorophyll content with the value of 0–99, which is determined by measuring leaf transmittance coefficients at 650 nm and 940 nm. The length and width of each leaf were measured to estimate the leaf area (Tan *et al.*, 2017), and LAI was calculated as the total leaf area divided by the projected land surface area of the canopy.

After harvest, the fresh weight of each corncob in the two treatments was measured. The dry matter and the mass of 100 randomly selected seeds (the 100-seed mass) were determined after corncobs had been oven-dried for 1 hour at 105 °C and 48 hours at 85 °C. The weights of oven-dried leaves, stems, and roots of three randomly selected plants in each treatment were also measured to explore the biomass allocation strategies of plants in response to water stress.

To explore the effects of leaf water levels on fluorescence parameters, in the early tasseling stage (65 days after sowing), after measurements of fluorescence parameters had been made on five fully expanded leaves of one randomly selected plant in each treatment, leaves were sampled and oven-dried for 1 hour at 105 °C and 48 hours at 85 °C. Leaf water content (LWC) was estimated as follows:

$$LWC=\frac{FW-DW}{FW}\times 100\%$$ (2)

where *FW* and *DW* are the fresh weight and dry weight, respectively, of each leaf.

### Measurement of chlorophyll fluorescence parameters

Chlorophyll fluorescence parameters (described in Table 2) were measured using an OS5P+ PAM fluorometer (Opti-Sciences Inc., Tyngsboro, MA, USA). Before measuring dark-adapted parameters, leaves were adapted to dark by applying leaf clips for 30 minutes (Liu *et al.*, 2012; Dobranszki and Mendler-Drienyovszki, 2014; Yang *et al.*, 2017). Then, the minimum and maximum fluorescence of dark-adapted leaves (F_o_ and F_m_, respectively) were measured with leaves exposed to modulated light [660 nm light-emitting diode (LED) light source with a 690 nm short pass filter, 0.5 μmol m^–2^ s^–1^] and saturated flash light (white LED with 690 nm short pass filter, 11250 μmol m^–2^ s^–1^ with a duration of 0.8 s), respectively. The maximum quantum yield of PSII of dark-adapted leaf (F_v_/F_m_) was then calculated by using the software in the fluorometer.

**Table 2.** Fluorescence parameters measured by the OS5P+ fluorometer

Parameter	Description
F_m_	Maximal fluorescence after dark adaptation
F_v_/F_m_	Maximum photochemical quantum yield under dark adaptation: F_v_/F_m_=(F_m_–F_o_)/F_m_ (Yang *et al.*, 2017), where F_o_ is the minimal fluorescence after dark adaptation
F_m_ʹ	Maximal fluorescence under the light condition
F_s_	Steady-state fluorescence
P yield	Actual quantum yield of PSII photochemistry, which indicates the proportion of light energy used in photochemistry by PSII under steady-state photosynthetic light conditions. P yield=(F_m_ʹ-F_s_)/F_m_ʹ (Genty *et al.*, 1989)
NPQ	Non-photochemical quenching, a process in which excess absorbed light energy is dissipated as heat. NPQ=(F_m_-F_m_ʹ)/F_m_ʹ (Tietz *et al.*, 2017)

After the measurement of dark-adapted parameters, the leaf clips were removed and the leaves were exposed to ambient light condition for 30 minutes. Then, light-adapted parameters were measured using the Yield protocol of the fluorometer. After leaves had been adapted in actinic light (white LED, 100 μmol m^–2^ s^–1^) for 5 minutes, fluorescence reached steady state (F_s_). Then, the maximum fluorescence of light-adapted leaves (F_m_ʹ) was measured by illumination with saturated flash light (the same as described above for dark-adapted leaves). The actual quantum yield of PSII (P yield) was then calculated from F_s_ and F_m_ʹ. Next, the light-adapted parameters under actinic light intensity of 500 μmol m^–2^ s^–1^ were measured.

Finally, rapid light response curves (RLCs) of F_s_ and F_m_ʹ were determined by the RLC protocol of the fluorometer with incident PAR programmed at values of 100, 150, 200, 250, 300, 350, 400, 450, 500, 600, and 700 μmol m^–2^ s^–1^, which took 2 minutes for each leaf. Parameters under dark and light conditions were measured at the same position for each leaf sample.

### Calculation of fluorescence yield and NPQ yield

A fluorometer was used to measure the P yield and the pulse signal intensity of F_s_, but it cannot directly determine the F_s_ yield. In order to explore the relationship between the two light-use efficiencies (i.e. F_s_ yield and P yield), the method proposed in the Soil-Canopy Observation of Photosynthesis and Energy model (van der Tol *et al*., 2014; Lee *et al.*, 2015; Zhang *et al.*, 2016*b*) was used to calculate the F_s_ yield. As heat dissipation is one of the three pathways that consumes the absorbed photons in the photosynthetic apparatus, the NPQ yield is achieved simultaneously as an auxiliary. In this method, heat dissipation is partitioned as the constitutive dark-adapted thermal dissipation (*d*) and the energy-dependent heat dissipation (*n*) under the light condition. The yields are calculated as follows, using the method described by Lee *et al.*, (2015), van der Tol *et al*., (2014), and Zhang *et al.* (2016*b*):

$$\Phi_{f}+\Phi_{p}+\Phi_{d}+\Phi_{n}=1$$ (3)

$$\Phi_{f}=\frac{K_{f}}{K_{f}+K_{d}+K_{p}+K_{n}}$$ (4)

$$\Phi_{p}=\frac{K_{p}}{K_{f}+K_{d}+K_{p}+K_{n}}$$ (5)

$$\Phi_{d}=\frac{K_{d}}{K_{f}+K_{d}+K_{p}+K_{n}}$$ (6)

$$\Phi_{n}=\frac{K_{n}}{K_{f}+K_{d}+K_{p}+K_{n}}$$ (7)

$$K_{d}=\max\left(0.03\times T+0.0773,\;0.87\right)$$ (8)

Kn=Fm−Fm'Fm'×(Kf+Kd) (9)

Kp=Fm'−FsFs×(Kf+Kd+Kn) (10)

where Φ is the probability of the fate of absorbed photons with subscripts *f* for fluorescence, *p* for photochemical reactions, and *n* and *d* for heat dissipation (the sum of $\Phi_{n}$ and $\Phi_{d}$ is the NPQ yield), respectively; $K_{f}$ is the rate constant for fluorescence, taken as 0.05 (van der Tol *et al*., 2014; Lee *et al.*, 2015); $K_{d}$ is the rate coefficient of dark-adapted thermal dissipation, which is a function of temperature (*T*) (van der Tol *et al*., 2014; Lee *et al.*, 2015); $K_{n}$ and $K_{p}$ are the rate coefficients of energy-dependent heat dissipation and photochemistry, respectively (van der Tol *et al*., 2014); $F_{m}$ is the maximal fluorescence after dark adaptation; Fm' is the maximal fluorescence under the light condition; and $F_{s}$ is the steady-state fluorescence. $\Phi_{p}$, $F_{m}$, Fm', and $F_{s}$ can all be measured directly by the OS5P+ fluorometer.

### Statistical analysis

Differences in measurements between the two water treatments, and the correlations between F_s_ yield, P yield, and NPQ yield, were analyzed. The *t*-test was used for comparisons between the mean values of measurements under the WS and NS conditions. As the time-series samples or variables changing with light intensity were not normally distributed and were paired across the two water treatments, the pairwise Wilcoxon non-parametric method was used to test differences in these parameters between the two treatments. To evaluate the relationship between two variables, regression analysis was performed with the coefficient of determination (*R*^2^) to describe its level of significance. Pearson and Spearman correlation coefficients were used for the linear and non-linear correlation analysis, respectively. Statistical significance was accepted when *P*<0.05.

## Results

### Effects of water stress on growth of summer maize

**Fig. 3.**
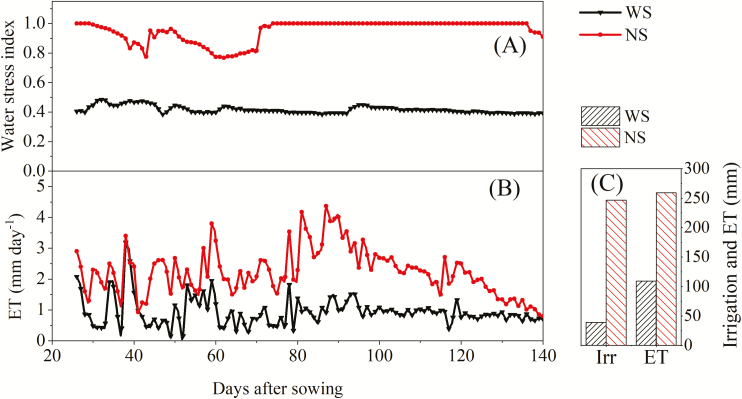
Variations in (A) water stress index and (B) evapotranspiration (ET); (C) total amount of irrigation (Irr) and ET during the whole growth period for the water stress (WS) and non-water stress (NS) treatments.

During the growth period, water stress indices for the NS treatment exceeded 0.77, whereas those for the WS treatment were lower, ranging from 0.38 to 0.48 (Fig. 3A). The irrigation amounts during the whole growth period were 246 mm for the NS treatment and 39 mm for the WS treatment (Fig. 3C). ET was significantly different between the two treatments (Fig. 3B); total ET during the whole growth period was 259 mm for the NS treatment and 109 mm for the WS treatment.

**Fig. 4.**
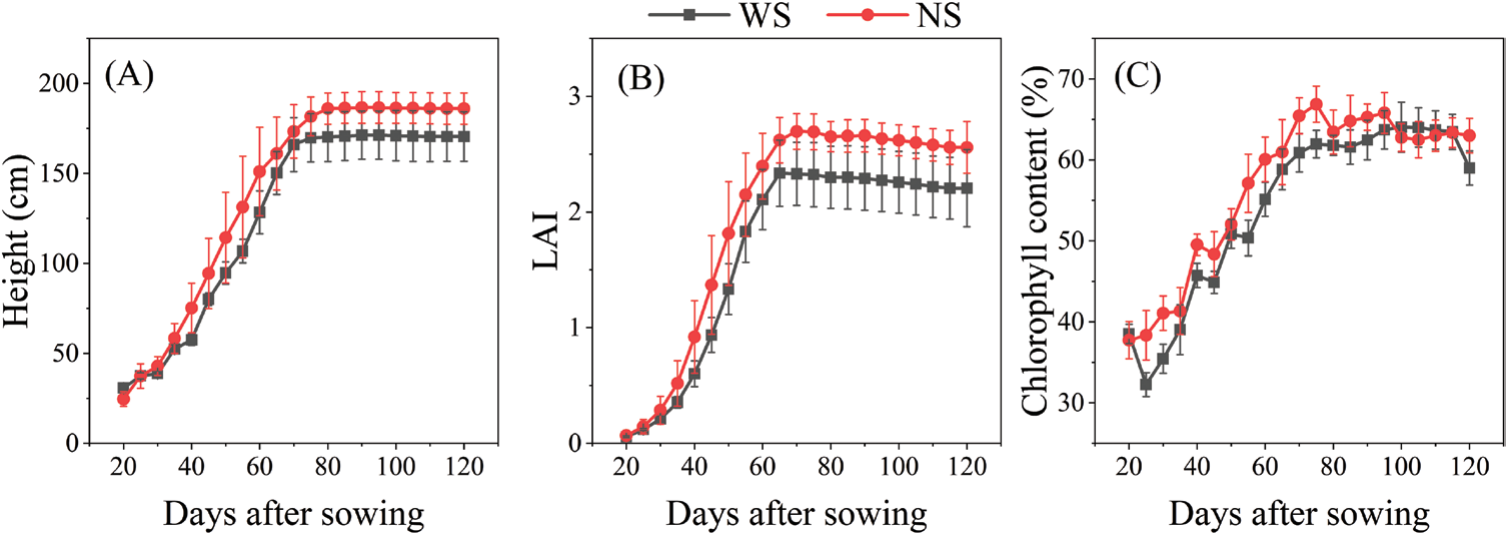
Mean height, (B) leaf area index (LAI), and (C) relative chlorophyll content of water-stressed (WS) and non-water stressed (NS) summer maize. Data points are mean values obtained from a set of five plant samples; error bars represent ±1 SD.

As shown in Fig. 4, there were significant differences between WS and NS plants in terms of plant height, LAI, and leaf chlorophyll content during the growth period (*P*<0.001 for height and LAI, *P*=0.001 for chlorophyll content). The maximum stalk heights of NS plants were significantly higher than those in the WS treatment (*P*=0.047), with mean values of 186.7 cm and 171.3 cm, respectively. The maximum LAI of plants in the NS treatment was significantly higher than that for the WS treatment (*P*=0.031).

**Fig. 5.**
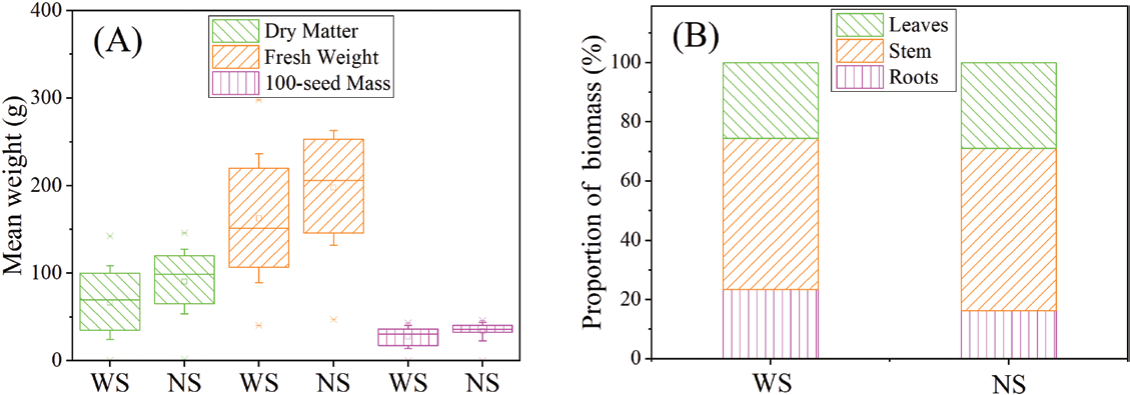
(A) Dry matter, fresh weight, and 100-seed mass per corncob (*n*=33; error bars represent ±1 SD); (B) mean proportions of biomass in the roots, stems, and leaves for the water stress (WS) and non-water stress (NS) treatments (*n*=3).

Significant differences between WS and NS treatments were also observed for indicators of crop yield (*P*=0.017 for dry matter, *P*=0.047 for fresh weight, and *P*=0.045 for 100-seed mass). Relative to the NS treatment, the mean dry matter of corncobs and the 100-seed mass in plants subjected to WS were reduced by 28.6% and 20.4%, respectively (Fig. 5A). The water use efficiency (defined here as crop yield divided by ET) of summer maize was 3.12 kg m^–3^ in the WS treatment and 1.95 kg m^–3^ in the NS treatment. The proportions of dry mass in the roots, stems, and leaves were 23.4%, 51.0%, and 25.6%, respectively, for plants in the WS treatment, and 16.2%, 54.9% and 28.9%, respectively, for plants in the NS treatment (Fig. 5B).

### Effects of water stress on fluorescence, photochemistry, and NPQ

**Fig. 6.**
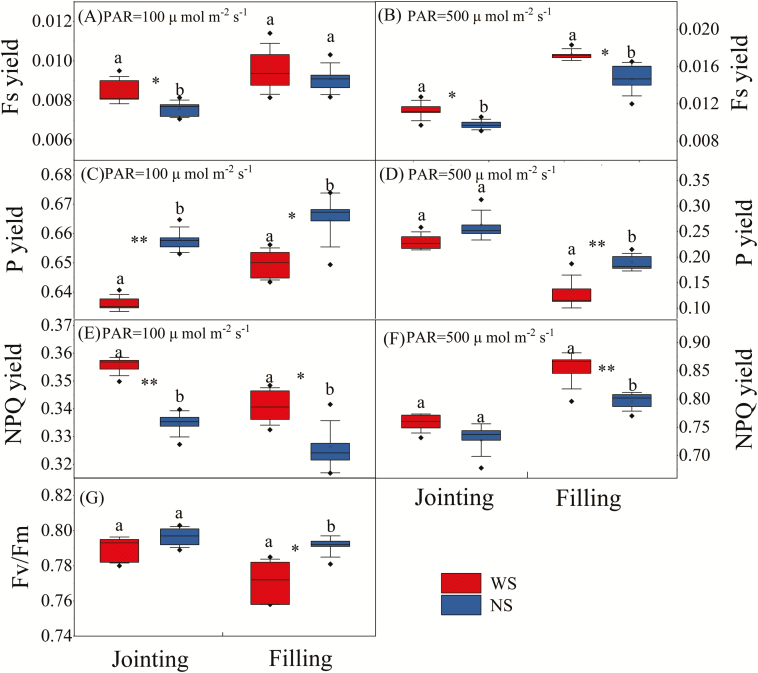
Comparisons of F_v_/F_m_, F_s_ yield, P yield, and NPQ yield in leaves of summer maize between the water stress (WS) and non-water stress (NS) treatments at the jointing stage (45 days after sowing) and filling stage (90 days after sowing) under (A, C, E) low light (PAR=100 μmol m^–2^ s^–1^) and (B, D, F) intermediate light (PAR=500 μmol m^–2^ s^–1^) conditions. Data are based on measurements made on five plants in each treatment. The top and bottom of the box represent the quartiles; the horizontal line in the box represents the median value; the whiskers represent ±1 SD. Mean values in the two treatments were compared by the *t*-test; different letters indicate significant differences: *p <0.05, **p<0.01.

In PSII, the absorbed light has three fates: the photochemical reaction, heat dissipation, and fluorescence emissions by chlorophyll (Porcar-Castell *et al.*, 2014; van der Tol *et al*., 2014). There are trade-offs between these three yields when plants acclimate to a changing environment (Baker, 2008). Fluorescence parameters measured in plants subjected to different water treatments and light conditions in two critical growth stages (the jointing and filling stages) are shown in Fig. 6. Significant differences in F_v_/F_m_ measurements between the two treatments were observed in the filling stage (*P*=0.013) but not in the jointing stage (Fig. 6G). This difference may be related to the smaller water deficit in the jointing stage than in the filling stage (Fig. 3A). Nevertheless, in both stages, the F_v_/F_m_ of WS leaves was lower than that of NS leaves, revealing the negative effects of water stress on the potential photon quantum yield. The actual P yields were also lower in the WS condition (Fig. 6C, D), whereas F_s_ yields increased under WS (Fig. 6A, B), indicating that more absorbed light was emitted as fluorescence. The NPQ yields in the two water treatments were consistent with those of F_s_ yields (Fig. 6E, F), because under the WS condition a higher proportion of energy was dissipated as heat to avoid damage resulting from excess light. The differences in P yields and NPQ yields between the two water treatments were significant in both the jointing and filling stages under the low light condition (PAR=100 μmol m^–2^ s^–1^; *P*<0.001 and *P*=0.023 for P yield in the jointing and filling stages, *P*<0.001 and *P*=0.023 for NPQ yield in the jointing and filling stages, respectively), but were significant only in the filling stage under the intermediate light condition (PAR=500 μmol m^–2^ s^–1^; *P*=0.008 and *P*=0.009 for P yield and NPQ yield, respectively). The F_s_ yields of the two water treatments showed significant differences in the jointing stage under the low light condition (*P*=0.033), and in both stages under the intermediate light condition (*P*=0.026 in the jointing stage and *P*=0.015 in the filling stage). Light intensity also affected the three yields. F_s_ yields and NPQ yields were significantly higher, while P yields were significantly lower, under the intermediate light condition (Fig. 6B, D, F) compared with the low light condition (Fig. 6A, C, E) (all *P* values <0.001).

**Fig. 7.**
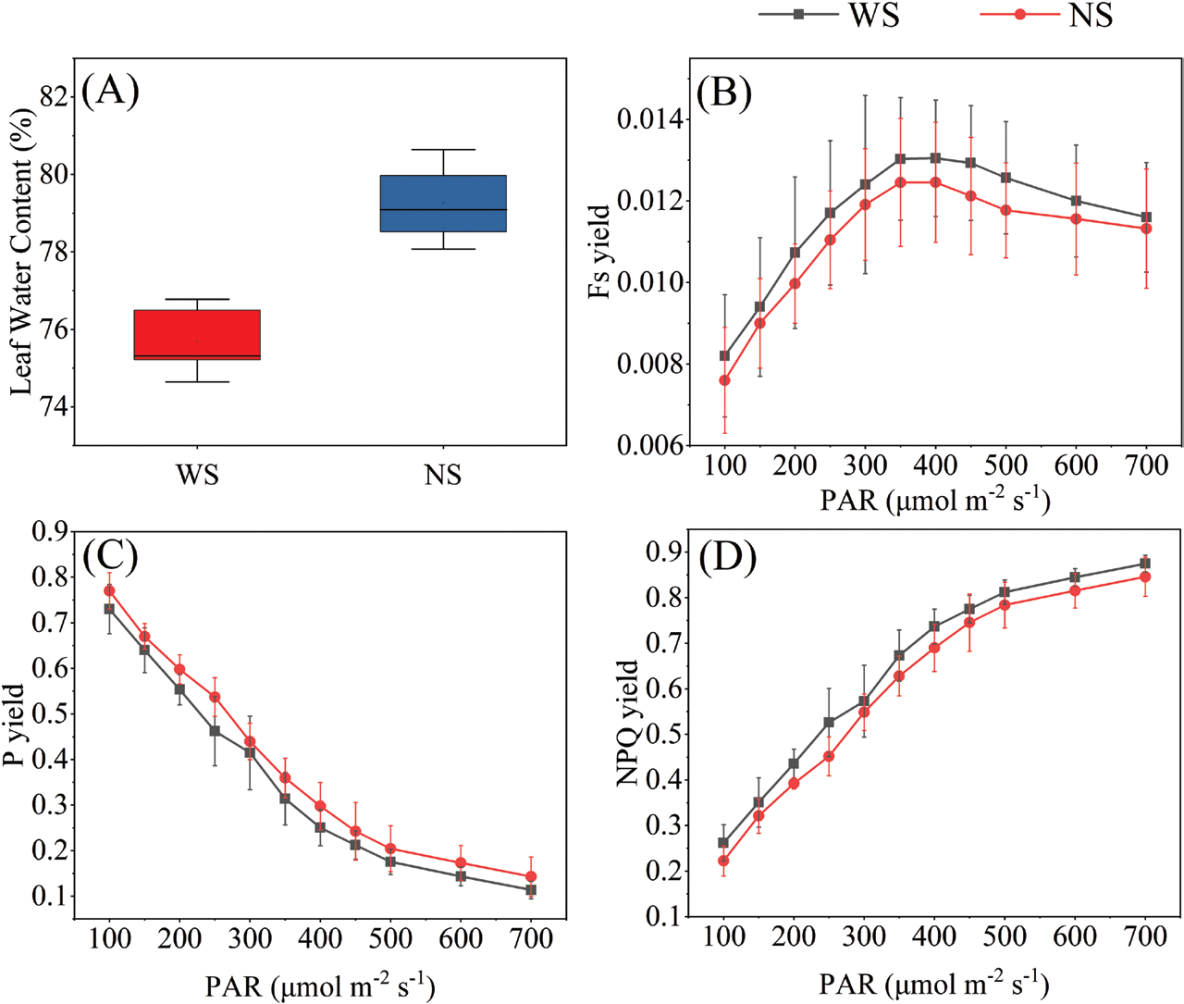
Leaf water contents and rapid light response curves of (B) F_s_ yield, (C) P yield, and (D) NPQ yield in the water stress (WS) and non-water stress (NS) treatments measured in the early tasseling stage (65 days after sowing). Data are based on measurements made on five fully expanded leaves on one plant in each treatment. Error bars represent ±1 SD.

In the early tasseling stage, five fully expanded leaves from plants subjected to each treatment were oven-dried to estimate LWC after measuring the fluorescence parameters. As shown in Fig. 7A, the differences in leaf water statuses between the two treatments were significant (*P*<0.001), with LWC being 75.7% for WS leaves and 79.3% for NS leaves. The rapid light response curves of F_s_ yield, P yield, and NPQ yield also showed significant differences between the WS and NS conditions (*P*=0.003 for all three yields). Under the WS condition, P yield was lower and NPQ yield and F_s_ yield were higher than the respective yields in the NS treatment (Fig. 7B–D). The P yield decreased with increasing PAR, indicating higher light use efficiency under low light levels. The increase of NPQ yield with increasing PAR indicated that more absorbed light was dissipated under higher light levels. The response of F_s_ yield to light intensity was not linear: F_s_ yield increased with PAR when PAR was <400 μmol m^–2^ s^–1^, and then decreased as PAR increased from 450 to 700 μmol m^–2^ s^–1^.

### Effects of water stress on the relationship between photochemical and fluorescence yields

**Fig. 8.**
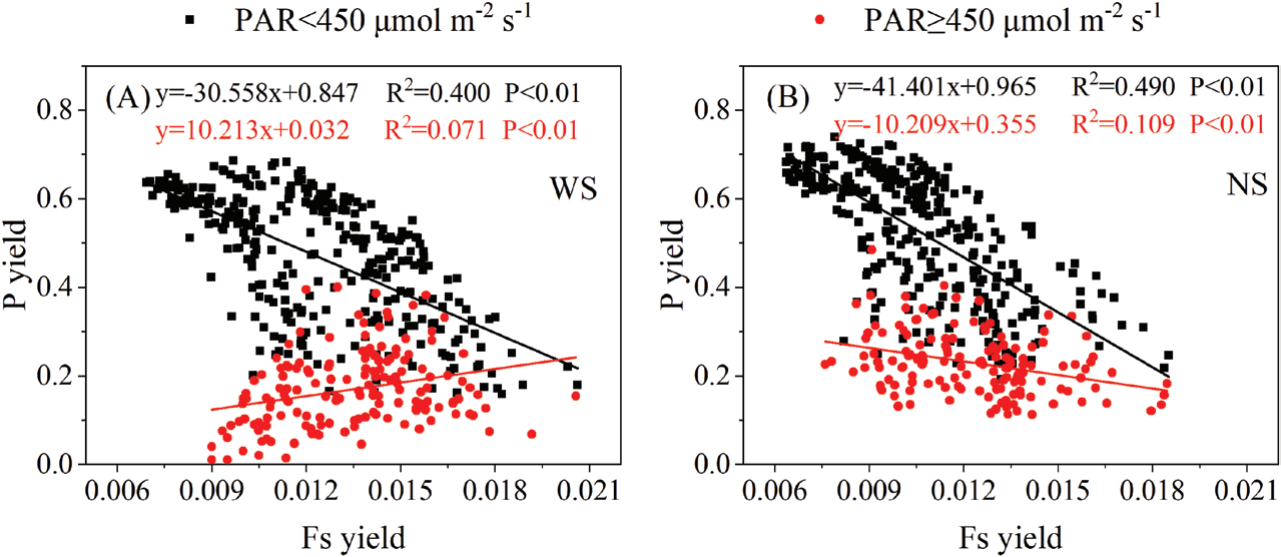
Relationships between F_s_ yield and P yield at PAR levels lower or higher than 450 μmol m^–2^ s^–1^ for the (A) water stress and (B) non-water stress treatments. The fit line and the corresponding equation are shown in the same colour.

As shown in Fig. 7B, there is a tipping point for the light response curve of F_s_ yield at light intensity ~450 μmol m^–2^ s^–1^. In view of this finding, we explored the relationships between F_s_ yield and P yield at light intensities lower and higher than 450 μmol m^–2^ s^–1^ (Fig. 8). We found that the correlation of F_s_ yield and P yield was negative under the low light condition (PAR <450 μmol m^–2^ s^–1^) for both the WS (*R*^2^=0.400, *P*<0.01) and NS (*R*^2^=0.490, *P*<0.01) treatments (Fig. 8). When PAR exceeded 450 μmol m^–2^ s^–1^, the relationships were different. P yield and F_s_ yield were still negatively related in the NS condition (*R*^2^=0.109, *P*<0.01), although the correlation was weaker. By contrast, for the WS treatment, the relationship was now positive (*R*^2^=0.071, *P*<0.01). Generally, under intermediate and low light conditions, higher light intensity causes the closure of photosynthetic reaction centers by reducing the primary quinone acceptor of PSII (Q_A_); consequently, photochemical quenching is reduced while fluorescence intensifies (Baker, 2008; Porcar-Castell *et al.*, 2014). Thus, F_s_ yield is negatively related to P yield. However, for the WS treatment, due to the dominant role of NPQ in dissipating excessive light (Flexas *et al.*, 2002; Liu *et al.*, 2012), F_s_ yield and P yield both decreased with increases of PAR above 450 μmol m^–2^ s^–1^ (Fig. 7), leading to a positive relationship between the two yields. Therefore, light intensity and water status are critical factors that comprehensively regulate the relationship between F_s_ yield and P yield.

**Fig. 9.**
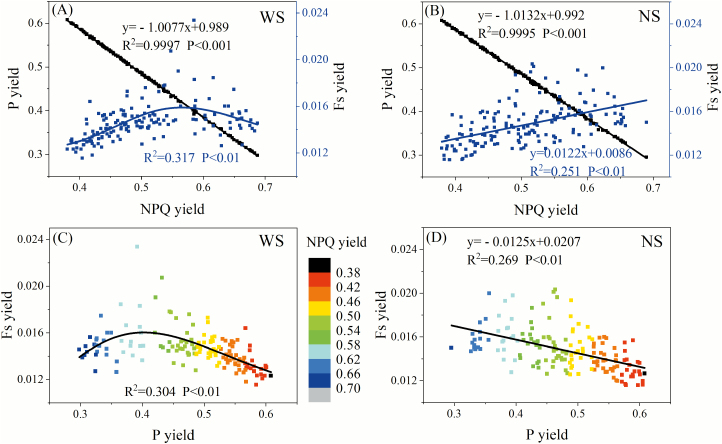
Relationships between F_s_ yield, P yield, and NPQ yield under low light conditions (PAR=100 μmol m^–2^ s^–1^). Blue symbols in (A) and (B) correspond to the right axis, and black symbols correspond to the left axis. The fit line and the corresponding equation are shown in the same color. Gradient colors in (C, D) denote the changes of NPQ yield.

The relationships between the yields of the three energy consumption pathways under low light conditions (PAR at 100 μmol m^–2^ s^–1^) were further explored. For the WS treatment, the NPQ yield was negatively and linearly related to the P yield (*R*^2^=0.9997, *P*<0.001) and non-linearly related to the F_s_ yield (*R*^2^=0.317, *P*<0.01) (Fig. 9A), resulting in a non-linear relationship between F_s_ yield and P yield (*R*^2^=0.304, *P*<0.01) (Fig. 9C). As shown in Fig. 9C, the F_s_ yield increased and then decreased as the P yield increased, along with the declining NPQ yield. When the P yield was below 0.40, NPQ played a dominant role in consuming light energy (NPQ yield >0.58), and the relationship between the F_s_ yield and P yield was positive. As the NPQ yield decreased (<0.58) and P yield increased (> 0.40), however, the F_s_ yield decreased and was negatively correlated with the P yield (Fig. 9C). Under the NS condition (Fig. 9B, D), the F_s_ yield and NPQ yield changed synergistically, and the P yield was negatively related to both the NPQ yield (*R*^2^=0.9995, *P*<0.001) and the F_s_ yield (*R*^2^=0.269, *P*<0.01).

## Discussion

### Effects of water stress on plant growth and photosynthetic physiology

Our study revealed that water stress reduced the proportion of dry matter contributed by stems and leaves, and increased that of roots (Fig. 5). Plants under water stress transferred more biomass to roots in order to take up water from deeper soil to relieve the stress (Yin *et al.*, 2005; Ge *et al.*, 2012). The lower proportion of the biomass attributable to leaves (or decreased LAI, as shown in Fig. 4) may also help the stressed plants adapt to the water-limited environment by reducing transpiration (Craufurd *et al.*, 1999). We also found that water use efficiency increased under the WS condition, as has also been reported in previous studies (Craufurd *et al.*, 1999; Yin *et al.*, 2005).

Water stress has a significant influence on plant photochemical and fluorescence characteristics (Flexas *et al.*, 2002; Faraloni *et al.*, 2011; Liu *et al.*, 2012; Cendrero-Mateo *et al.*, 2015). The three fluorescence parameters (F_s_, P yield, and NPQ) can effectively identify water stress, as suggested by previous studies (Flexas *et al.*, 2000; Souza *et al.*, 2004; Mishra *et al.*, 2012). In this experiment, summer maize grown under intermediate light conditions and subjected to moderate water stress and non-water stress treatments showed significant differences in their photosynthetic physiology: the P yield decreased and the F_s_ yield and NPQ yield increased in response to water stress.

P yield is directly related to photosynthesis since it quantifies the light-use efficiency of PSII. It is sensitive to stress and decreases under drought conditions (Lu and Zhang, 1999; Liu *et al.*, 2012; Mishra *et al.*, 2012). The reduction of P yield associated with water stress has been reported in tomato (Mishra *et al.*, 2012), maize (Liu *et al.*, 2012), cowpea (Souza *et al.*, 2004), wheat (Lu and Zhang, 1999), grapevine (Flexas *et al.*, 2000), and aloe (Hazrati *et al.*, 2016). In these studies, the decrease of P yield was accompanied by an increase in NPQ. We found similar results in the present study. The physiological mechanism for the decreased P yield and the increased NPQ yield of C_4_ plants under water stress can be explained as follows. Water stress induces stomatal closure (Flexas *et al.*, 2002; Rosales *et al.*, 2012; Mathobo *et al.*, 2017; Ouyang *et al.*, 2017); as a consequence, the intercellular CO_2_ concentration decreases, inducing a decline in the rate of carboxylation (Baker and Rosenqvist, 2004; Souza *et al.*, 2004). This results in feedback on the photoreaction to reduce electron use efficiency and retard electron transport (Baker, 2008; van der Tol *et al*., 2014). Then, more photosynthetic reaction centers are closed because reduced Q_A_ cannot be reoxidized in time, resulting in the inhibition of photochemical quenching and an increase in fluorescence (Baker, 2008). In addition, when the electron transport chain is saturated, protons accumulate and the thylakoid lumen pH decreases, which may amplify NPQ (Porcar-Castell *et al.*, 2014). In this case, electron and light energy are excessive (Flexas *et al.*, 2002), and fluorescence and NPQ are utilized to consume the excessive light. This explains the observed increase of F_s_ yield in summer maize (a C_4_ crop) under moderate water stress in this study.

The response of F_s_ to water stress in C_3_ plants can also help us understand the results observed in this study. Decreased fluorescence in C_3_ plants in response to water stress has been reported (Flexas *et al.*, 2000, 2002; Cendrero-Mateo *et al.*, 2015). For example, Flexas *et al*. (1999, 2000, 2002) reported that F_s_ or F_s_/F_o_ of grapevines decreased under water stress, which may result from photorespiration (Flexas *et al.*, 2000; Flexas and Medrano, 2002; Porcar-Castell *et al.*, 2014). Photorespiration increases as the CO_2_ concentration reduces due to stomatal closure under water stress (Flexas *et al.*, 2000). Photorespiration consumes NADPH (triphosphopyridine nucleotide) (Flexas *et al.*, 2000), which promotes electron transfer from reduced Q_A_ to the secondary electron acceptor and thus quenches fluorescence (Baker, 2008). Therefore, the fluorescence of C_3_ plants decreases under water stress. However, when the photorespiration of C_3_ plants was inhibited by exposing leaves to high CO_2_ concentrations in the absence of O_2_, the decrease of F_s_/F_o_ under water stress was suppressed and an increase of F_s_/F_o_ in response to water stress was observed with PAR <600 μmol m^–2^ s^–1^ (Flexas *et al.*, 2002). This observation indicates that fluorescence may increase under water stress in intermediate and low light conditions when there is no photorespiration. This is consistent with observations made in this study. In addition, Cendrero-Mateo *et al.* (2015) showed that the F_s_ yield of C_3_ plants under water stress decreased significantly at PAR of 300 or 500 μmol m^–2^ s^–1^, but it increased (albeit not significantly) when PAR was <300 μmol m^–2^ s^–1^. In the present study, the increase of F_s_ yield in response to moderate water stress was also observed for summer maize grown under intermediate and low light conditions (0–700 μmol m^–2^ s^–1^).

### Relationships between photochemistry, fluorescence, and heat dissipation

Chlorophyll fluorescence is an effective indicator for accurately estimating gross photosynthesis (Flexas *et al.*, 2002; Baker and Rosenqvist, 2004; Damm *et al.*, 2010; Lee *et al.*, 2013; van der Tol *et al*., 2014; Lee *et al.*, 2015). Some studies have found that fluorescence and photosynthesis are positively related (Guanter *et al.*, 2014; Cendrero-Mateo *et al.*, 2015). For example, the CO_2_ assimilation rate is positively related to F_s_ measured by PAM in leaves of *Camelina sativa* (Cendrero-Mateo *et al.*, 2015). Guanter *et al.* (2014) used a positive linear relationship to directly estimate global GPP by solar-induced fluorescence. However, a negative relationship between fluorescence and photosynthetic light use efficiency or P yield has also been reported (Damm *et al.*, 2010; Liu and Cheng, 2010). Liu and Cheng (2010) stated that photosynthetic light use efficiency is negatively related to chlorophyll fluorescence due to their competition for light energy. In PSII, light energy is partitioned between photochemistry, heat dissipation, and chlorophyll fluorescence (Baker, 2008; Cendrero-Mateo *et al.*, 2015; Guo *et al.*, 2015). Under non-stress conditions, most light energy is channeled into photochemistry, with smaller amounts of energy destined for heat dissipation and fluorescence emission (Porcar-Castell *et al.*, 2014). In the presence of stress, more energy is dissipated to relieve the stress, but the trade-off between, and changes in, the three pathways are still unclear.

Recently, studies have reported that the relationship between GPP and fluorescence is affected by physiological and ecological factors, such as vegetation type (Damm *et al.*, 2015; Guan *et al.*, 2016), land surface temperature (Cui *et al.*, 2017), irradiation (Zhang *et al.*, 2016*b*; Cui *et al.*, 2017), LAI, and chlorophyll content (Zhang *et al.*, 2016*b*; Cui *et al.*, 2017). This study revealed the relationship between fluorescence and photochemistry and the changes in this relationship depending on water status and light intensity. Light intensity is a crucial factor regulating the photosynthetic physiological processes (Flexas *et al.*, 2002; van der Tol *et al.*, 2009; Cendrero-Mateo *et al.*, 2015; Zhang *et al.*, 2016*b*; Cui *et al.*, 2017). Under low light conditions (PAR<450 μmol m^–2^ s^–1^), NPQ was inhibited by the proton gradient (Losciale *et al.*, 2011; Porcar-Castell *et al.*, 2014) and F_s_ yield increased, while P yield decreased with increasing light intensity (Fig. 7); in this case, F_s_ was negatively related to photochemistry. However, under water stress and intermediate light conditions of 450–700 μmol m^–2^ s^–1^, as the light intensity increased, the electron transport chain became saturated (as mentioned above). NPQ increased as protons accumulated and the thylakoid lumen pH decreased (Porcar-Castell *et al.*, 2014). In this case, fluorescence and photochemistry were both inhibited (Baker, 2008; Porcar-Castell *et al.*, 2014), resulting in a positive relationship between F_s_ yield and P yield (Fig. 8). Similar results have been reported in previous studies based on model simulations (van der Tol *et al.*, 2009; Damm *et al.*, 2015; Lee *et al.*, 2015).

We found that light energy used for photochemical reactions was negatively related to that emitted as fluorescence in the NS condition (Fig. 9). For leaves under the WS condition, we inferred that when NPQ took up a large proportion of the absorbed light energy, fluorescence and photochemistry were concurrently inhibited, resulting in a positive relationship between F_s_ yield and P yield. By contrast, when NPQ was not the primary light consumption pathway, the output between fluorescence and photochemistry changed. Together, these factors mean that the relationship between F_s_ yield and P yield for water-stressed plants was non-linear. Similar phenomena have been observed in previous research (Damm *et al.*, 2010; Liu and Cheng, 2010; Porcar-Castell *et al.*, 2014). For example, Porcar-Castell *et al.* (2014) reported that F_s_ yield is negatively related to P yield when NPQ is low, but the relationship becomes positive with increasing NPQ. Damm *et al.* (2010) illustrated that F_s_ yield may be positively or negatively related to photosynthetic light use efficiency, and that the slope of their relation function changes across different growing stages. Liu and Cheng (2010) also illustrated that fluorescence and photosynthesis have a competitive relationship only when NPQ is not operating. Baker (2008) and Damm *et al.* (2010) suggested that the principal inverse relationship between the energy used for fluorescence and that used for primary photosynthesis is usually lost due to the dominant role of NPQ in dissipating the excess light. Thus, it can be concluded that when NPQ is low, fluorescence emission is one of the processes that dissipate the excess light. In this case, F_s_ yield and P yield are inversely correlated. However, when NPQ plays a dominant role in dissipating the excess energy, F_s_ yield and P yield are both suppressed by NPQ yield; as a consequence, they are synergistic and positively related. Owing to complex relationships between fluorescence and photochemistry under changing environmental conditions, an increase in fluorescence may be associated with either an increase or a decrease in photochemistry. Thus, knowledge of the ambient environment is a prerequisite for assessing photochemistry by fluorescence (van der Tol *et al.*, 2009).

## Conclusion

Chlorophyll fluorescence is closely related to photosynthesis. To explore the relationship between photochemical yield and fluorescence yield, and the response of the relationship to water stress under intermediate light conditions, we conducted an experiment in a climate chamber. Summer maize grown under an above-canopy light intensity of 700 μmol m^–2^ s^–1^ was subjected to moderate water stress, with soil water content being 45–50% of the field capacity, and non-water stress, with soil water content of >80% of the field capacity.

Water stress has a significant influence on plant photosynthetic physiology. For plants grown in intermediate light conditions, the F_s_ yield and NPQ yield increase and the P yield decreases in response to water stress. Light intensity and water status affect the apportioning of light energy between heat dissipation, fluorescence, and photochemistry. Specifically, in the absence of water stress, F_s_ yield is negatively related to P yield in intermediate and low light conditions, regardless of the changes in NPQ yield. However, under water stress, the relationship between F_s_ yield and P yield is negative in low light conditions (PAR <450 μmol m^–2^ s^–1^) but positive in intermediate light conditions (PAR ≥450 μmol m^–2^ s^–1^ and ≤700 μmol m^–2^ s^–1^); F_s_ yield is negatively related to P yield when NPQ is <0.58, but when NPQ is >0.58, F_s_ yield and P yield are synergistically and positively associated.

These results reveal that in intermediate and low light conditions there are no single positive or negative relationships between P yield and F_s_ yield; the relationship between these yields may depend on light intensity and water status, and these factors should be considered when using measurements of chlorophyll fluorescence to estimate plant production.
